# Tram-track cycling injuries: a significant public health issue

**DOI:** 10.1007/s11845-022-03254-w

**Published:** 2023-01-09

**Authors:** Olivia Smith, Catherine McCabe, Emer Kidney

**Affiliations:** 1 Emergency Department, St James’s Hospital, Dublin, Ireland; 2 Trinity College, Dublin, Ireland; 3 Connolly Hospital Blanchardstown, Dublin, Ireland

**Keywords:** Cyclists, Emergency Department, Injuries, Luas, Tram-tracks

## Abstract

**Aim:**

Many cycling collisions occur due to human error, cycling ability, distraction or infrastructure. One such infrastructural issue for cyclists sharing the road with tram lines is where the wheel of the bicycle gets caught in the rail track itself or in a gap between the rail and the road margin resulting in a sudden stall of the bicycle and potentially significant injury. This study aims to describe the crash characteristics of tram-track cycling collisions and their associated injuries.

**Methods:**

A retrospective chart review was conducted over 2 years, looking at cyclists that presented to St James’s Emergency Department (ED) following injuries sustained due to a bicycle wheel catching in the on-road tram tracks.

**Results:**

Forty-eight patients were identified over a 2-year period. Sixty per cent of cyclists sustained limb fractures with 14% requiring orthopaedic surgery. Fifty per cent of patients were not wearing a helmet at the time of the incident and 54% of the collisions occurred around Dublin city centre during rush hour.

**Conclusion:**

Further prospective multi-centre studies are required to properly describe the magnitude cycling accidents around the Luas tracks and inform future public health measures in this area.

## Introduction

In Ireland, cyclists account for 14% of injuries from all road collisions. In 2008, figures show 336 reported cyclist collisions, increasing to 1058 in 2018 [1]. A recent Australian study found that hospitalised cyclists from single bicycle crashes accounted for nearly half of all road crashes, with 19% of those involving the cyclists bicycle wheel getting caught in an on-road tram-track [2]. These specific types of cycling crashes involving road infrastructure are frequently reported in international studies [3–7]. Such collisions appear to mainly occur while cyclists attempt to ride closely alongside the track line or cross it at an acute angle as opposed to crossing the track line at the recommended a 90-degree angle [4]. In Dublin city, many cycling forums (dublincycling. com, cycling.ie) as well as other media outlets (Dublin Inquirer/ Irish Times) have over the years anecdotally highlighted cycling crashes from around the Luas tram tracks but no formal study has been conducted. The aim of this study is to describe the crash characteristics and patient demographics for a group of patients attending a city centre emergency department (ED) after a bicycle crash where the Luas tracks were a contributing factor.

## Methods

We conducted a retrospective cohort study of cycling injuries related to the Luas tracks that presented to a single, city centre ED, St. James’s ED, between November 2017 and November 2019. Patients were identified at registration, at triage and during clinical consultation using convenience sampling techniques. Inclusion criteria were all adult patients who were injured in a cycling accident at or near the Luas tracks. Exclusion criteria were patients aged under 18, and patients in whose crashes the Luas tracks were not a contributory factor.

Patient notes and ambulance sheets were then interrogated to identify patient demographics, crash location and time and injury characteristics. Data analysis was conducted using the Statistical Package for the Social Sciences (SPSS) descriptive statistics software (www.ibm.com).

## Results

### Demographic

Forty-eight patients were identified as having presented with injuries specifically sustained when their bicycle wheel got caught in a tram track. Fifty-five per cent of patients were male and 45% female. Fifty per cent of patients were under the age of 30 years old, and 50% were over 30 years old, with an age range in the study of 20–60 years.

### Time

**Fig. 1:**
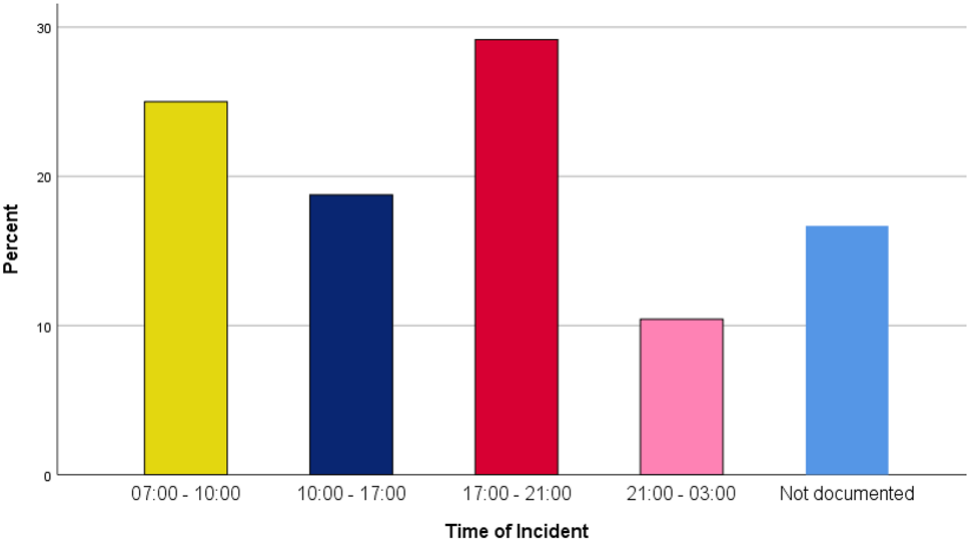
Time of incident occurring

As shown in Fig. 1, the majority of accidents (29.2%) occurred in evening rush hour (17.00–21.00) followed by (25%) occurring during morning rush hour (07.00–10.00). 41.7% of patients were brought to the ED by ambulance from the site of the accident with other less common modes of arrival including walking, private car, taxi and Dublin bus.

### Injury profile

Of the incidents that occurred, 60.4% (*n* = 29) of the patients sustained limb fractures and just under 21% (*n* = 10) sustained head injuries (Table 1). Upper limb injuries accounted for 56% (*n* = 27) of all injuries, and lower limb injuries were just over 33% (*n* = 16) of all incidents. Just over 8% (*n* = 4) of patients were admitted directly from the ED for orthopaedic surgery with a further 6.25% (*n* = 3) admitted for orthopaedic surgery from the fracture clinic.

**Table 1:** Injuries

**Presenting injury**	**Quantity**
Knee	8
Ankle	6
Hand	7
Wrist	4
Elbow	7
Shoulder	9
Chest	3
Head	10
Hip (pedal embedded in soft tissues)	1
Femur	1
Face/mouth	6

Some patients had a combination of two or more of the injuries shown in Table 1

Twenty-five per cent of patients had soft tissue injuries. Some were minor that were treated and discharged. Others required primary wound closure (23% of all patients) or follow-up with physio/OT or dressing clinic. Some of the more severe injuries included one patient with a fractured neck of femur and another with a part of their bicycle embedded in the soft tissues of the thigh.

Thirty-three per cent of patients in the study sustained a head injury. Fifty per cent (*n* = 24) of the patients were not wearing a cycling helmet at the time of the incident. Of note data on this variable was not captured in over 20% of cases so this figure may be higher.

### Location of incidents

Specific street locations were identified with the most significant number 45.8% (*n* = 22) of the incidents occurring at or near Trinity College in the city centre. 12.5% occurred on Stephens Lane in Dublin 8 near Heuston train station with a similar number 12.5% occurring on Dawson Street in the city centre. Other separate locations around the city centre amounted to 27.1% of accidents recorded.

## Discussion

This audit demonstrates that cycling in and around tram track lines or crossing tram track lines in Dublin city has inherent dangers. Accidents occurred as cyclists crossed the tracks or when the wheel slipped into the track space as they cycled adjacent to the line. From the resulting fall, 60% of the patients in this study sustained a limb fracture 14% of which required orthopaedic surgery. These type injuries have the potential to not only impact significantly on the day-to-day lives of patients and their employment capability [8-9], but also at a psychological level on injury outcome and ability to return to work [10].

While 33% of patients sustained head trauma in the incident, it appears cyclists are not heeding road safety messages regarding the wearing of a helmet with over 50% of patients in this study not wearing a helmet at the time of the incident. Bil et al. [11] identified that head trauma and intracranial haemorrhages among cyclists could be reduced by the wearing of a helmet while cycling, particularly and specifically in low energy falls and tumbles from a bicycle or a bicycle striking an obstacle, which are similar to those captured in this study. These specific low velocities and low-to-medium trauma groups were highlighted as the cycling group within which wearing a helmet could be the most beneficial. Gerber et al. [12] report similar injuries and also highlight the associated high resource needs, particularly radiological work-up.

This study found that there were definitive “cycling black spots” within the city, where cycling incidents crossing the Luas tracks were most frequent. The area around College Green in the city centre had highest number of incidents. It may be that the area where the rail phalanges intersect warrants closer examination and scrutiny, with possible engineering solutions developed to avoid further injury in these areas. It does appear and is generally advised that cyclists need to cross tram tracks at a direct 90-degree angle rather than at an acute angle. This method of track-crossing would reduce the risk of the wheel getting caught in the track. However, in busy rush hour traffic when most incidents did occur, accurate manoeuvring across tram tracks may prove challenging or impossible at traffic-clogged intersections.

Filling in of the grooves has been suggested but appears to present engineering difficulties [13]. Awareness of tyre depth versus rail width is also critical. The manoeuvrability of city bikes versus owner racing bikes, for example, may also be a factor and could be explored in future studies. Better signage to heighten awareness among cyclists of the dangers of cycling over tracks in these areas may also need to be considered.

This audit relates to just one of six adult EDs in Dublin city. It should be noted that many cyclists who are involved in incidents near Luas tracks may not have received medical attention at all, or they may have attended their general practitioner or a private minor injuries unit or one of the other ED’s in the city, rather than St. James’s Emergency Department. It is possible too that not all presenting incidents may have been captured. Therefore, it is possible the number of cyclists involved in tram track cycling injuries in Dublin city may be significantly higher than the number of incidents identified here.

## Conclusion

Further multi-centre prospective studies using a systematic sampling technique and including follow-up of cyclists involved in such incidents in order to analyse the real effect of tram track injuries on livelihood, occupation and employers should be conducted. Resource issues for the city from such incidents may be significant. The cost of emergency ambulance call-out to the scene, together with the hospital resources needed to provide care, treatments and follow-up, needs to be investigated. Enhancing cyclist education concerning sharing the streets with tram lines may help to reduce accidents as may adopting preventative measures at known or suspected accident blackspots. As previously mentioned, other studies have highlighted that similar cycling collisions have been identified in other cities with on road tram lines. Further prospective multi-centre studies may show similar results and highlight the need for action both on an individual basis and at a systems level in order to avoid such injuries and possibly more catastrophic outcomes for cyclists in the future.

## Data Availability

No official data available on Luas cycling injuries from other EDs in Dublin City.
